# Supplementary material for: PLK1 inhibition delays mitotic entry revealing changes to the phosphoproteome of mammalian cells early in division
Source: EMBO J. 2025 Mar 3;44(7):1891–920. doi: 10.1038/s44318-025-00400-9 (PMC11962124; doi:10.1038/s44318-025-00400-9)
Supplement: Supplementary file 1 — Appendix [file 44318_2025_400_MOESM1_ESM.pdf]

Appendix for:

PLK1 inhibition delays mitotic entry revealing changes to the phosphoproteome of mammalian cells early in division

Table of Contents:

Page 2: Appendix Figure S1

Page 3: Legend for Appendix Figure S1

Page 4: Appendix Table S1

Page 5: Legend for Appendix Table S1

Appendix Figure S1.

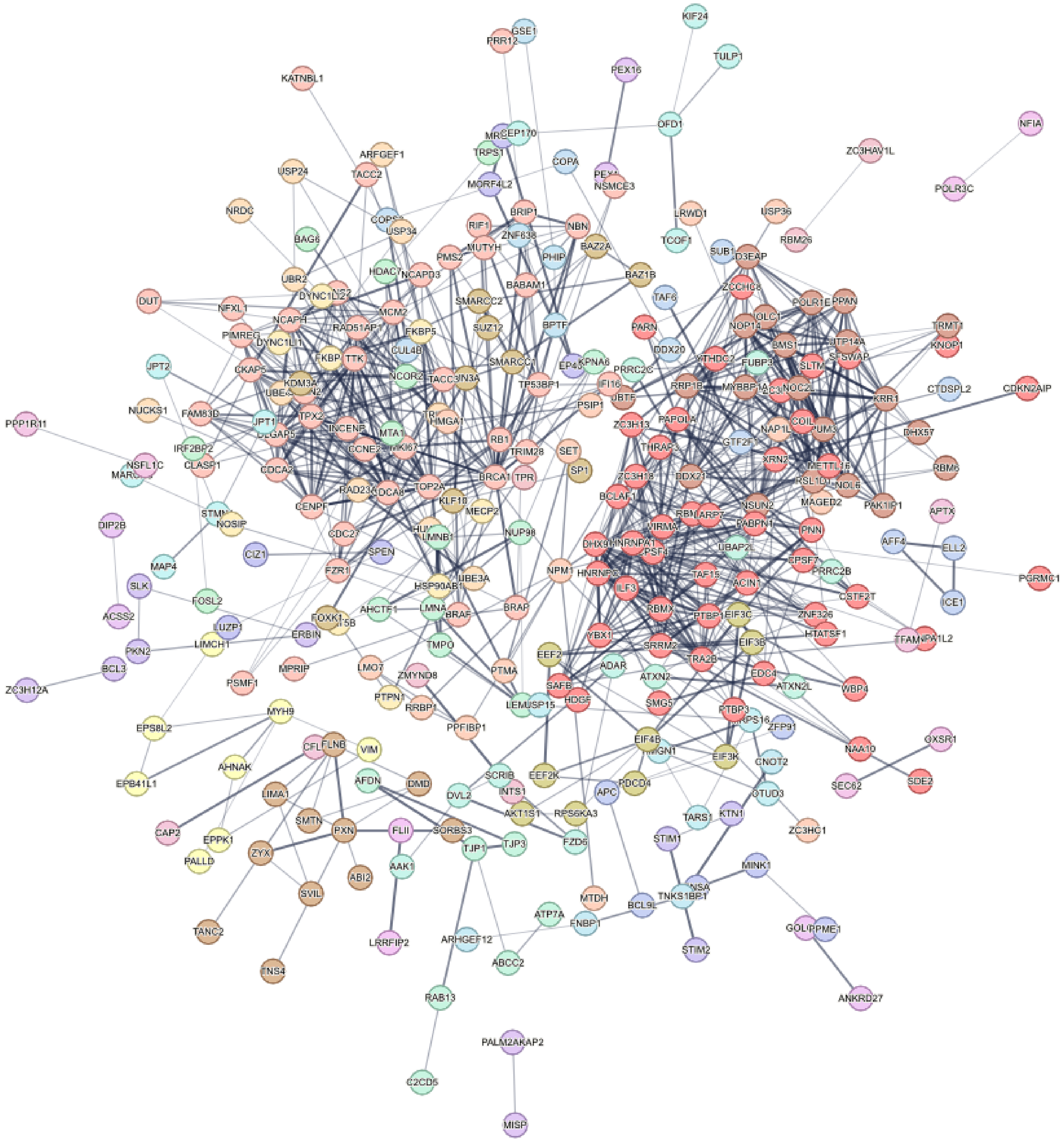

Appendix Figure S1:

Network generated by STRING database analysis depicting differentially phosphorylated proteins specific to prolonged prophase after applying linear modeling. The gene names of the corresponding proteins are shown.

Appendix Table S1.

Gene name	Protein name	Position	Sequence window	Log2 Fold Change	Adjusted p value
BABAM1	BRISC and BRCA1-A complex member 1	29	EPRPRTRSNPEGAED	-0.13564	0.452216
		65	ADDGSLNTSGAGPKS	3.204965	0.016133
BRAF	Serine/threonine-protein kinase B-raf	429	PQRERKSSSSEDRN	0.020957	0.950661
		732	HRSASEPSLNRAGFQ	-0.46754	0.021532
BRAP	BRCA1-associated protein	97	ERKSSEASPTAQRSK	0.758572	0.036624
		571	IAMASASSPASSGGS	1.233362	0.15329
BRCA1	Breast cancer type 1 susceptibility protein	1524	LQNRNYPSQEELIKV	-1.30686	0.007531
		967	GNETGLITPNKHGLL	-0.17774	0.826994
BRIP1	Fanconi anemia group J protein	1032	SSENSASSPPRFKTE	0.035918	0.946208
		226	CSTKQGNSQESSNTI	-1.83983	0.001085
		990	IVISRSTSPTFNKQT	-0.23038	0.223636
CCNE2	G1/S-specific cyclin-E2	21	PQPSQTESPQEAQII	-2.4767	0.000111
CDC27	Cell division cycle protein 27 homolog	220	NRLNLESSNSKYSLN	-1.35576	0.004893
CDCA2	Cell division cycle-associated protein 2	710	EPKAGTDSPVSCASV	1.182501	0.000399
CDCA8	Borealin	219	YNISGNGSPLADSKE	1.338073	0.001152
		154	PSKKRTQSIQGKGKG	0.468087	0.752849
CENPF	Centromere protein F	821	CRLEADQSPKNSAIL	0.501219	0.225936
		2922	SVTEKRLSSGQNKAS	0.573635	0.094435
		1121	MTDNQNNSKSEAGGL	3.917572	0.001717
		3079	VNNLPERSPTDSPRE	2.153058	0.000459
		3054	KVKVAQRSPVDSGTI	0.114245	0.720904
		144	VSLNPCNTPQKIFTT	0.911767	0.011247
CKAP5	Cytoskeleton-associated protein 5	816	FEKMQGQSPPAPTRG	1.182614	0.044594
CLASP1	CLIP-associating protein 1	1223	SRDGGAASPATEGRG	0.340762	0.124086
		1091	SSNTSVGSPSNTIGR	1.696642	0.040077
		646	RIRTRRQSSGSATNV	0.307958	0.44256
		656	SATNVASTPDNRGRS	1.075414	0.101246
DLGAP5	Disks large-associated protein 5	662	GIPQQTTSPENAGPQ	2.025834	0.000199
		777	KNTASQNSILEEGET	0.407237	0.715857
DUT	Deoxyuridine 5'-triphosphate nucleotidohydrolase, mitochondrial	248	RGSGGFGSTGKN___	1.260963	0.002084
FAM64A	Protein FAM64A	106	RIQESCQSGTKWLVE	1.82903	0.009454
		16	GTSVRRRSLQHQEQL	0.592557	0.057232
FAM83D	Protein FAM83D	462	GTQSTEGSPVSKMSV	1.241782	0.001824
FZR1	Fizzy-related protein homolog	70	RINENEKSPSQNRKA	0.825274	0.00045
GINS2	DNA replication complex GINS protein PSF2	182	QPLESTQSQDF____	-1.61545	0.001327
IFI16	Gamma-interferon-inducible protein 16	106	KKEVDATSPAPSTSS	0.976518	0.002982
		153	EEQTQPPSPAGAGMS	-0.22222	0.496304
		123	KTEGAEATPGAQKRK	-0.25215	0.559768
INCENP	Inner centromere protein	446	GPREPPQSARRKRSY	-0.0091	0.988528
		263	KLRIAQVSPGPRDSP	0.986793	0.150242
		421	NPKPAASSPETPSAG	1.649486	0.031907
		481	PRSKTPSSPCPASKV	0.066519	0.826603
KATNBL1	KATNB1-like protein 1	135	SGSSQTESPSSKYSG	0.816669	0.007063
MCM2	DNA replication licensing factor MCM2	13	ESFTMASSPAQRRRG	-0.20928	0.503571
		139	RRGLLYDSDEEDEER	-0.19919	0.461216
		27	GNDPLTSSPGRSSRR	-1.03073	0.026971
MKI67	Antigen KI-67	584	VISPPAPSPRKTPVA	0.204023	0.909901
		2783	PAASVTGSRRRPRAP	1.784411	0.031297
		1253	TTKIPCDSPQSDPVD	1.950414	0.00924
		1329	LTENLTGSKRRPQTP	0.812784	0.592089
		1815	QPGNLPGSNRRLQTR	-0.13797	0.787722
		2022	VGKLTQTSGKTTQTH	1.108794	0.020309
		357	QYSQQQNSPQKHKNK	-0.04028	0.939795
		1376	TKMPCESSPPESADT	1.319096	0.005691
		374	YTTGRRESVNLGKSE	-0.17389	0.739664
		648	MICSKRRSGASEANL	1.680698	0.24483
		621	PKRGGRKSGNLPSKR	1.771414	0.027648
		1628	DPDKNPASSKRRLKT	0.515792	0.524267
		328	RDVESVQTPSKAVGA	0.014021	0.984762
		2773	LKESAKQTPAPAASV	0.50022	0.7761
		1327	LDLTENLTGSKRRPQ	0.454921	0.213038
		1503	SQPDPVDTPTSSKPQ	0.396177	0.455166
MPRIP	Myosin phosphatase Rho-interacting protein	891	GGGEATGSPLAQGKD	1.011246	0.001912
MUTYH	A/G-specific adenine DNA glycosylase	512	SKRSQVSSPCSRKKP	0.420998	0.023535
NBN	Nibrin	343	TTPGPSLSQGVSVDE	-1.53502	0.000459
NCAPD3	Condensin-2 complex subunit D3	517	FSYQRQTSNRSEPSG	-1.86704	0.02044
NCAPH	Condensin complex subunit 2	15	LPATMNNSSSETRGH	2.490922	0.032082
		459	KQDAPSQSENKKKST	0.078348	0.943523
		87	DSPRLLASPSSRSID	0.861254	0.38164
NDNL2	Melanoma-associated antigen G1	304	PSGPAPSS_______	1.001316	0.008161
		64	SQGSQGPSPQGARRA	1.064173	0.029366
NFXL1	NF-X1-type zinc finger protein NFXL1	50	GAVPSGTSPGGVATT	1.595657	0.033303
PMS2;PMS2CL	Mismatch repair endonuclease PMS2;Protein PMS2CL	403	MVEKQDQSPSLRTGE	1.193663	0.00045
PRR12	Proline-rich protein 12	917	AGAYRSPSPQGTKAP	-1.10341	0.015456
PSMF1	Proteasome inhibitor PI31 subunit	153	WEKANVSSPHREFPP	1.074455	0.003203
RAD51AP1	RAD51-associated protein 1	120	VTTNVQNSQDKSIEK	-1.65658	0.004118
		327	LVVVSVKSPNQSLRL	0.582974	0.183504
RB1	Retinoblastoma-associated protein	249	AVIPINGSPRTPRRG	0.77095	0.007063
		356	DSFETQRTPRKSNLD	0.240314	0.15906
RIF1	Telomere-associated protein RIF1	2161	SLVSANDSPSGMQTR	0.736809	0.003275
		1579	WKNKSNESVDIQDQE	0.03069	0.925885
		1688	KRLHKRDSFDNCSLG	-0.41527	0.187504
		2195	RSQEDEISSPVNKVR	0.319566	0.418184
		2196	SQEDEISSPVNKVRR	0.215008	0.539879
SLAIN2	SLAIN motif-containing protein 2	63	IPSSGAASPRGFPLG	1.578614	0.000635
		48	AGSLGPGSPVRAGAS	0.000119	0.999812
TACC2	Transforming acidic coiled-coil-containing protein 2	1635	PRSTCAPSPQREVLT	1.402082	0.008431
		2256	DSGGQEDSPAKGLSV	0.519993	0.036132
		2226	GGGRVQNSPPVGRKT	-0.17854	0.215832
TACC3	Transforming acidic coiled-coil-containing protein 3	317	AGRAMTLSPQEEVAA	0.369546	0.423235
		434	SEAQPPESPETRLGQ	0.822245	0.001201
TOP2A	DNA topoisomerase 2-alpha	1106	DEEENEESDNEKETE	-0.05939	0.862834
		1247	KNENTEGSPQEDGVE	0.102269	0.858992
		4	____MEVSPLQPVNE	0.940392	0.003275
		1213	QMAEVLPSPRGQRVI	1.924142	7.36E-05
TP53BP1	Tumor suppressor p53-binding protein 1	862	TQEKTSNSLTEDSKM	1.845217	0.104161
		552	IEDTEPMSPVLNSKF	-2.26502	0.003153
		1028	AVAESVASPQKTMSV	-0.50497	0.007574
		831	EPVEQDSSQPSLPLV	-0.28053	0.416727
		380	TPFIVPSSPTEQEGR	-0.57136	0.008651
TPX2	Targeting protein for Xklp2	186	TAEKNASSPEKAKGR	0.140839	0.41381
		738	QPLTVPVSPKFSTRF	1.107704	0.000479
		369	KICRDPQTPVLQTKH	0.074426	0.84967
TRIM28	Transcription intermediary factor 1-beta	19	AASAASGSPGPGEGS	-0.29259	0.514772
		697	TGVVAKLSPANQRKC	0.771591	0.088797
		473	SGVKRSRSGEGEVSG	-0.38443	0.018897
TTK	Dual specificity protein kinase TTK	436	VFSVSKQSPPISTSK	0.464797	0.018196

Appendix Table S1:

Table showing all proteins depicted in Fig. 6A, with the corresponding gene names, position of phosphosites detected, sequence window around the phosphosites and the results of enrichment in prolonged prophase after applying linear modeling for analysis.
