# Supplementary material for: PLK1 inhibition delays mitotic entry revealing changes to the phosphoproteome of mammalian cells early in division
Source: EMBO J. 2025 Mar 3;44(7):1891–920. doi: 10.1038/s44318-025-00400-9 (PMC11962124; doi:10.1038/s44318-025-00400-9)
Supplement: Supplementary file 2 — Table EV1 [file 44318_2025_400_MOESM2_ESM.docx]

**Table EV1:** Parameter values for the model of Rata et al. 2018, used for the simulations shown in Figure 1, 2 and EV2. Compared to the original work we rescaled all kinetic parameters, except those indicated in bold, by a factor of 0.025 to account for the duration of G2-phase of several hours (right column). The total cdk2-cyclin A concentration is varied to model the differences between cells.

| **Name** | **Name in code** | **Description** | **Value** | **Ratio to parameters in Rata et al. (2018)** |
| --- | --- | --- | --- | --- |
| [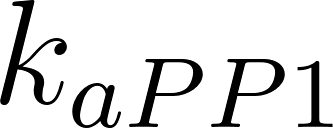](https://www.codecogs.com/eqnedit.php?latex=k_%7BaPP1%7D#0) | kapp1 | Constitutive dephosphorylation and thereby activation of PP1 | 0.01725 [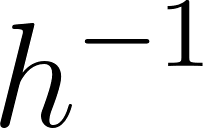](https://www.codecogs.com/eqnedit.php?latex=h%5E%7B-1%7D#0) | 0.025 |
| [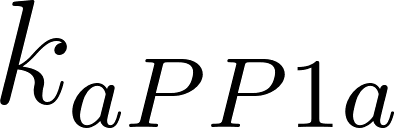](https://www.codecogs.com/eqnedit.php?latex=k_%7BaPP1a%7D#0) | kapp1a | Dephosphorylation of PP1 by dephosphorylated PP1 in trans | 1.0581 [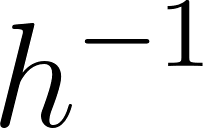](https://www.codecogs.com/eqnedit.php?latex=h%5E%7B-1%7D#0) | 0.025 |
| [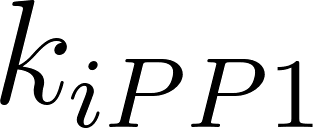](https://www.codecogs.com/eqnedit.php?latex=k_%7BiPP1%7D#0) | kipp1 | Constitutive phosphorylation and hence inactivation of PP1 | 0.0027 [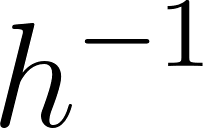](https://www.codecogs.com/eqnedit.php?latex=h%5E%7B-1%7D#0) | 0.025 |
| [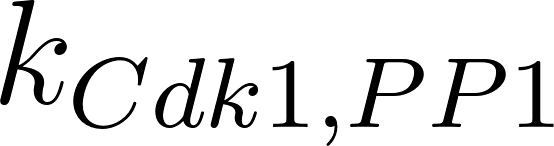](https://www.codecogs.com/eqnedit.php?latex=k_%7BCdk1%2CPP1%7D#0) | kipp1C | Phosphorylation of PP1 by Cdk1 | 1.1323 [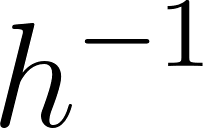](https://www.codecogs.com/eqnedit.php?latex=h%5E%7B-1%7D#0) | 0.025 |
| [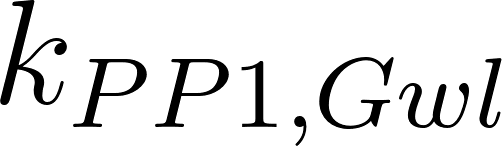](https://www.codecogs.com/eqnedit.php?latex=k_%7BPP1%2CGwl%7D#0) | kPP1Gw | Dephosphorylation of Gwl by PP1 | 27.7086 [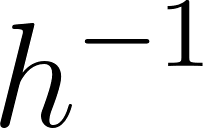](https://www.codecogs.com/eqnedit.php?latex=h%5E%7B-1%7D#0) | 0.025 |
| [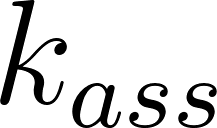](https://www.codecogs.com/eqnedit.php?latex=k_%7Bass%7D#0) | kass | Association of pENSA with PP2A:B55 | 925.9211 [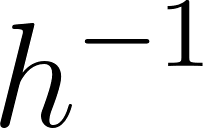](https://www.codecogs.com/eqnedit.php?latex=h%5E%7B-1%7D#0) | 0.025 |
| [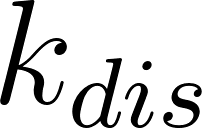](https://www.codecogs.com/eqnedit.php?latex=k_%7Bdis%7D#0) | kdis | Dissociation of the pENSA:PP2A:B55 complex | 0.0132 [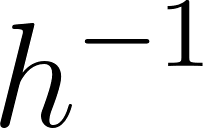](https://www.codecogs.com/eqnedit.php?latex=h%5E%7B-1%7D#0) | 0.025 |
| [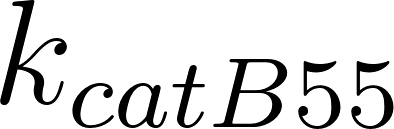](https://www.codecogs.com/eqnedit.php?latex=k_%7BcatB55%7D#0) | kcatB55 | Dephosphorylation of pENSA when in complex with PP2A:B55 | 1.5507 [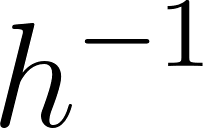](https://www.codecogs.com/eqnedit.php?latex=h%5E%7B-1%7D#0) | 0.025 |
| [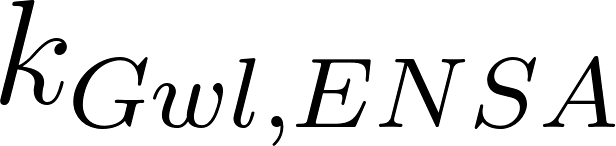](https://www.codecogs.com/eqnedit.php?latex=k_%7BGwl%2CENSA%7D#0) | kGwENSA | Phosphorylation of ENSA by Gwl | 31.3216 [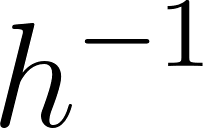](https://www.codecogs.com/eqnedit.php?latex=h%5E%7B-1%7D#0) | 0.025 |
| [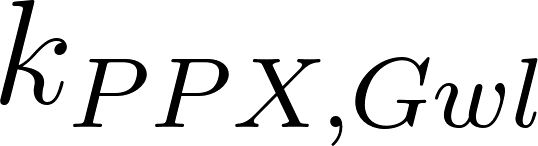](https://www.codecogs.com/eqnedit.php?latex=k_%7BPPX%2CGwl%7D#0) | kppxGwl | Basal dephosphorylation of Gwl | 0.234 [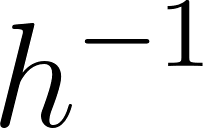](https://www.codecogs.com/eqnedit.php?latex=h%5E%7B-1%7D#0) | 0.025 |
| [**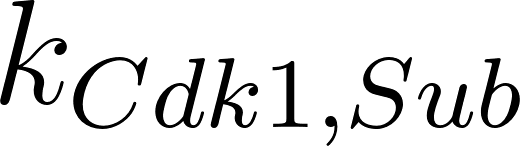**](https://www.codecogs.com/eqnedit.php?latex=k_%7BCdk1%2CSub%7D#0) | **kcBc1Sub** | **Phosphorylation of the substrate by Cdk1** | **0.12** [**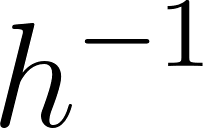**](https://www.codecogs.com/eqnedit.php?latex=h%5E%7B-1%7D#0) | **0.250** |
| [**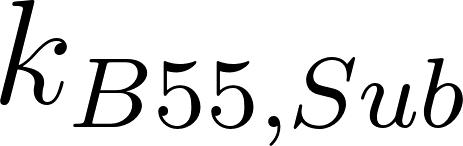**](https://www.codecogs.com/eqnedit.php?latex=k_%7BB55%2CSub%7D#0) | **kB55Sub** | **Dephosphorylation of the substrate by PP2A:B55** | **0.8895** [**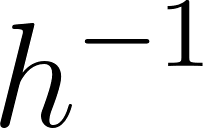**](https://www.codecogs.com/eqnedit.php?latex=h%5E%7B-1%7D#0) | **0.250** |
| [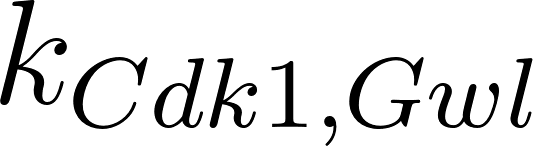](https://www.codecogs.com/eqnedit.php?latex=k_%7BCdk1%2CGwl%7D#0) | kcBc1G | Phosphorylation of Gwl by Cdk1 | 0.35895 [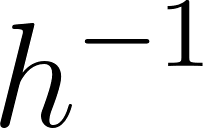](https://www.codecogs.com/eqnedit.php?latex=h%5E%7B-1%7D#0) | 0.025 |
| [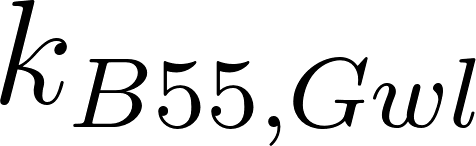](https://www.codecogs.com/eqnedit.php?latex=k_%7BB55%2CGwl%7D#0) | kB55G | Dephosphorylation of Gwl by PP2A:B55 | 744.8454 [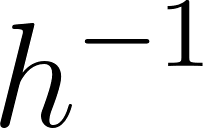](https://www.codecogs.com/eqnedit.php?latex=h%5E%7B-1%7D#0) | 0.025 |
| [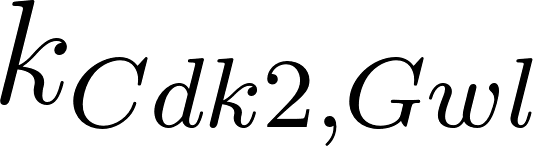](https://www.codecogs.com/eqnedit.php?latex=k_%7BCdk2%2CGwl%7D#0) | kcAc2G | Phosphorylation of Gwl by Cdk2 | 0.2874 [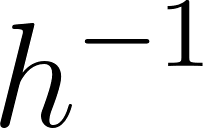](https://www.codecogs.com/eqnedit.php?latex=h%5E%7B-1%7D#0) | 0.025 |
| [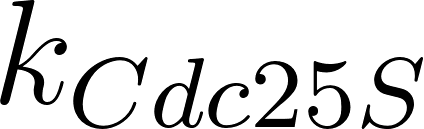](https://www.codecogs.com/eqnedit.php?latex=k_%7BCdc25S%7D#0) | k25S | Dephosphorylation of Y15 of Cdk1 by unphosphorylated Cdc25 | 0.0075 [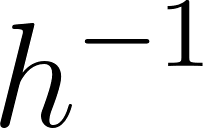](https://www.codecogs.com/eqnedit.php?latex=h%5E%7B-1%7D#0) | 0.025 |
| [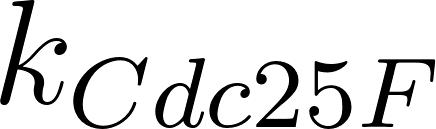](https://www.codecogs.com/eqnedit.php?latex=k_%7BCdc25F%7D#0) | k25F | Dephosphorylation of Y15 of Cdk1 by phosphorylated Cdc25 | 1.4117 [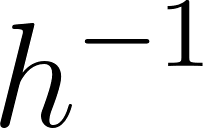](https://www.codecogs.com/eqnedit.php?latex=h%5E%7B-1%7D#0) | 0.025 |
| [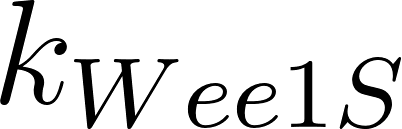](https://www.codecogs.com/eqnedit.php?latex=k_%7BWee1S%7D#0) | kweeS | Phosphorylation of Y15 of Cdk1 by phosphorylated Wee1 | 0.0075 [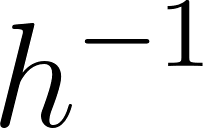](https://www.codecogs.com/eqnedit.php?latex=h%5E%7B-1%7D#0) | 0.025 |
| [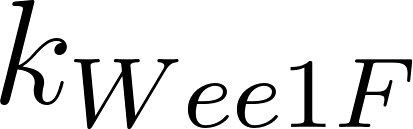](https://www.codecogs.com/eqnedit.php?latex=k_%7BWee1F%7D#0) | kweeF | Phosphorylation of Y15 of Cdk1 by unphosphorylated Wee1 | 70.9406 [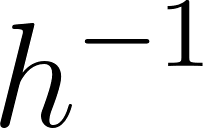](https://www.codecogs.com/eqnedit.php?latex=h%5E%7B-1%7D#0) | 0.025 |
| [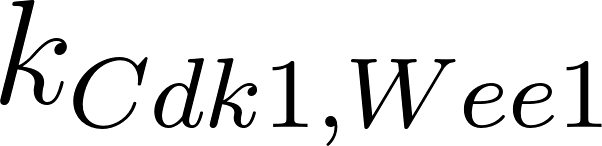](https://www.codecogs.com/eqnedit.php?latex=k_%7BCdk1%2CWee1%7D#0) | kcBc1W1 | Phosphorylation of Wee1 by Cdk1 | 1.9698 [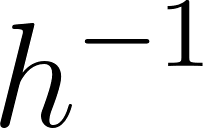](https://www.codecogs.com/eqnedit.php?latex=h%5E%7B-1%7D#0) | 0.025 |
| [**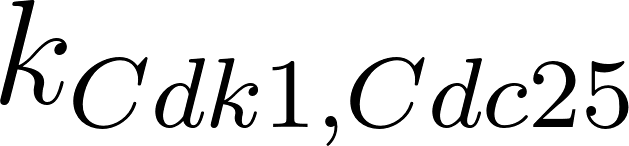**](https://www.codecogs.com/eqnedit.php?latex=k_%7BCdk1%2CCdc25%7D#0) | **kcBc125** | **Phosphorylation of Cdc25 by Cdk1** | **0.19698** [**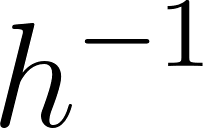**](https://www.codecogs.com/eqnedit.php?latex=h%5E%7B-1%7D#0) | **0.0025** |
| [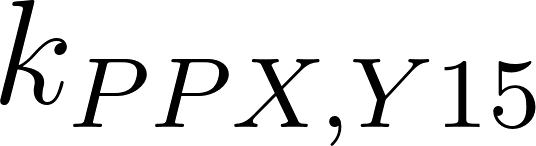](https://www.codecogs.com/eqnedit.php?latex=k_%7BPPX%2CY15%7D#0) | kppxY15 | Dephosphorylation of Wee1 and Cdc25 by a constitutive phosphatase | 0.0075 [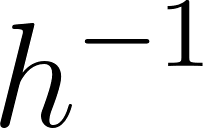](https://www.codecogs.com/eqnedit.php?latex=h%5E%7B-1%7D#0) | 0.025 |
| [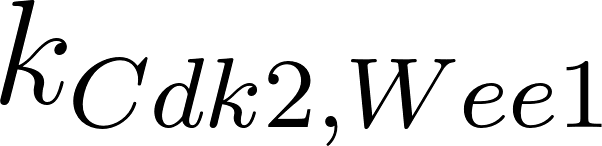](https://www.codecogs.com/eqnedit.php?latex=k_%7BCdk2%2CWee1%7D#0) | kcAc2W1 | Phosphorylation of Wee1 by Cdk2 | 0.1644 [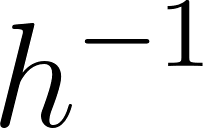](https://www.codecogs.com/eqnedit.php?latex=h%5E%7B-1%7D#0) | 0.025 |
| [**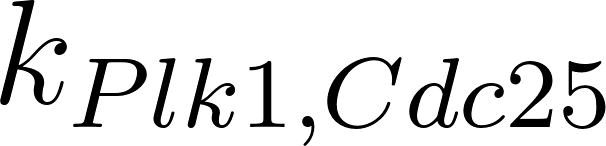**](https://www.codecogs.com/eqnedit.php?latex=k_%7BPlk1%2CCdc25%7D#0) | **kplk1225** | **Phosphorylation of Cdc25 by Plk1** | **0.01644** [**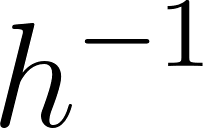**](https://www.codecogs.com/eqnedit.php?latex=h%5E%7B-1%7D#0) | **0.0025** |
| [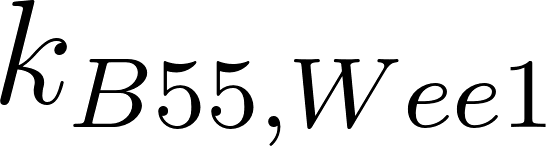](https://www.codecogs.com/eqnedit.php?latex=k_%7BB55%2CWee1%7D#0) | kB55W1 | Dephosphorylation of Wee1 by PP2A:B55 | 0.82665 [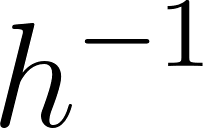](https://www.codecogs.com/eqnedit.php?latex=h%5E%7B-1%7D#0) | 0.025 |
| [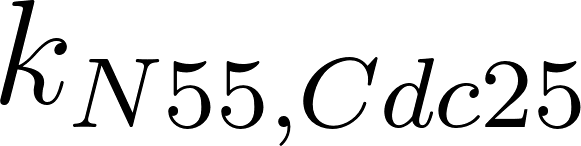](https://www.codecogs.com/eqnedit.php?latex=k_%7BN55%2CCdc25%7D#0) | kB5525 | Dephosphorylation of Cdc25 by PP2A:B55 | 0.82665 [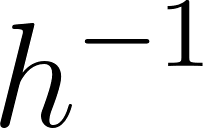](https://www.codecogs.com/eqnedit.php?latex=h%5E%7B-1%7D#0) | 0.025 |
| [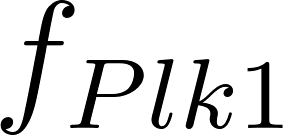](https://www.codecogs.com/eqnedit.php?latex=f_%7BPlk1%7D#0) | fplk1 | Fraction active Plk1 | 0 - 1 | NA |
| [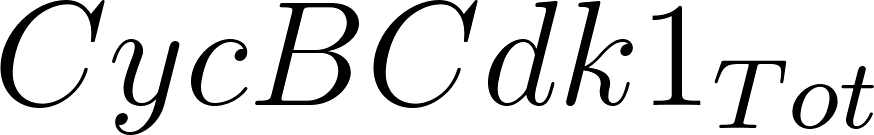](https://www.codecogs.com/eqnedit.php?latex=CycBCdk1_%7BTot%7D#0) | CycBCdk1T | Total Cdk1 Cyclin B complexes | 8.1808 a.u. | 1 |
| [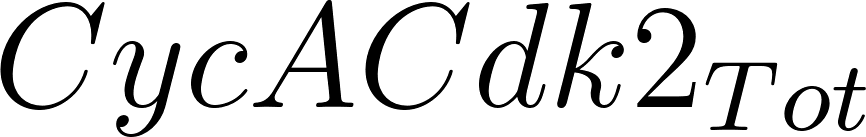](https://www.codecogs.com/eqnedit.php?latex=CycACdk2_%7BTot%7D#0) | CycACdk2T | Total Cdk2 Cyclin A complexes | 2 - 5 a.u. | 2-5 |
| [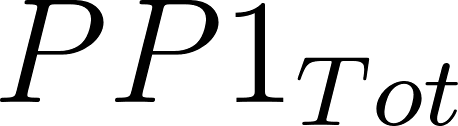](https://www.codecogs.com/eqnedit.php?latex=PP1_%7BTot%7D#0) | PP1T | Total PP1 | 1 a.u. | 1 |
| [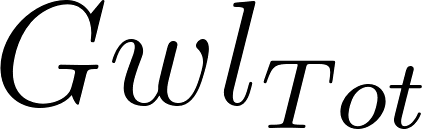](https://www.codecogs.com/eqnedit.php?latex=Gwl_%7BTot%7D#0) | Gwtot | Total Gwl | 1 a.u. | 1 |
| [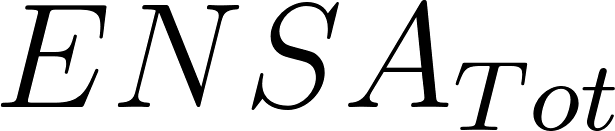](https://www.codecogs.com/eqnedit.php?latex=ENSA_%7BTot%7D#0) | ENSAtot | Total Ensa | 1 a.u. | 1 |
| [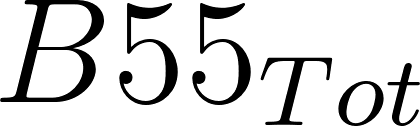](https://www.codecogs.com/eqnedit.php?latex=B55_%7BTot%7D#0) | B55tot | Total B55 | 0.25 a.u. | 1 |
| [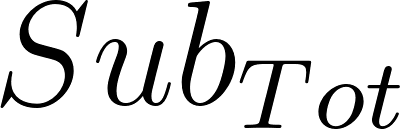](https://www.codecogs.com/eqnedit.php?latex=Sub_%7BTot%7D#0) | SubT | Total Cdk1 phosphorylation substrate | 1 a.u. | 1 |
