# Supplementary figures and images for: PLK1 inhibition delays mitotic entry revealing changes to the phosphoproteome of mammalian cells early in division
Source: EMBO J. 2025 Mar 3;44(7):1891–920. doi: 10.1038/s44318-025-00400-9 (PMC11962124; doi:10.1038/s44318-025-00400-9)

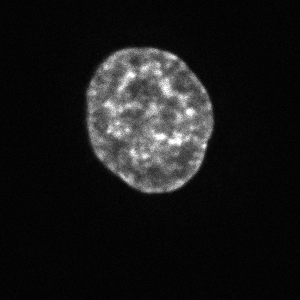

Supplement: Supplementary file 7 — Source data Fig. 1 [file 44318_2025_400_MOESM7_ESM.zip › Figure 1/1B/20211130_C6_2_DMSO.tif]

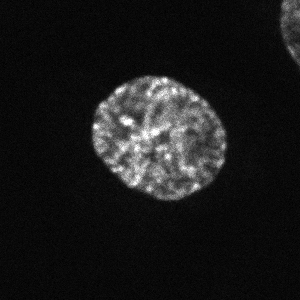

Supplement: Supplementary file 7 — Source data Fig. 1 [file 44318_2025_400_MOESM7_ESM.zip › Figure 1/1B/20211130_F5_3_BI2536.tif]

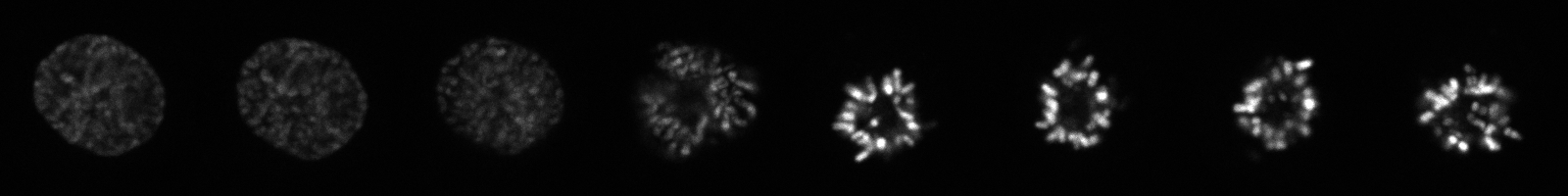

Supplement: Supplementary file 7 — Source data Fig. 1 [file 44318_2025_400_MOESM7_ESM.zip › Figure 1/1B/Cell division 250 20211130 montage 10umscale.tif]

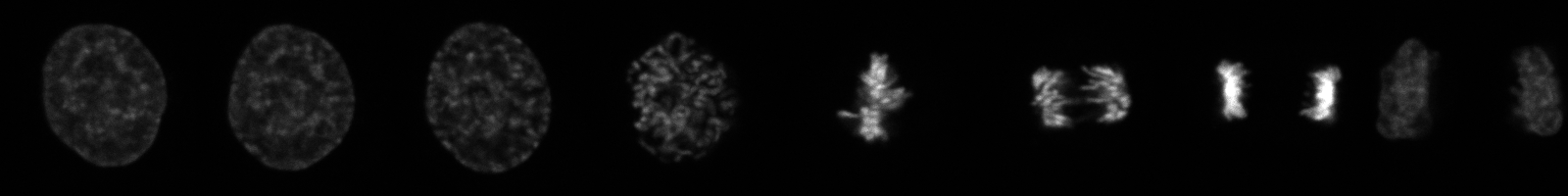

Supplement: Supplementary file 7 — Source data Fig. 1 [file 44318_2025_400_MOESM7_ESM.zip › Figure 1/1B/Cell division Ctrl 20211130 montage 10umscale.tif]

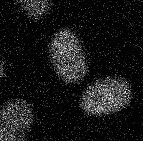

Supplement: Supplementary file 8 — Source data Fig. 2 [file 44318_2025_400_MOESM8_ESM.zip › Figure 2/2A/BI2536_MAX_20230816_A2_-1.tif]

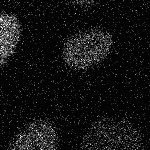

Supplement: Supplementary file 8 — Source data Fig. 2 [file 44318_2025_400_MOESM8_ESM.zip › Figure 2/2A/DMSO_MAX_20230816_A1_-1.tif]

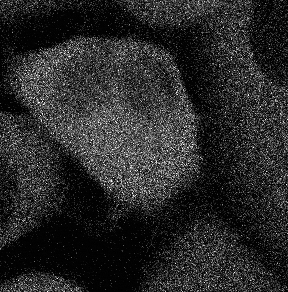

Supplement: Supplementary file 8 — Source data Fig. 2 [file 44318_2025_400_MOESM8_ESM.zip › Figure 2/2B/BI2536_MAX_20231018_C4_-1.tif]

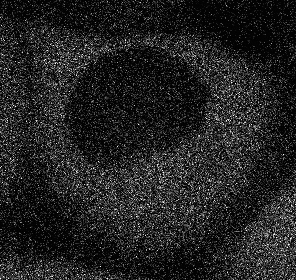

Supplement: Supplementary file 8 — Source data Fig. 2 [file 44318_2025_400_MOESM8_ESM.zip › Figure 2/2B/DMSO_MAX_20231018_C3_-1.tif]

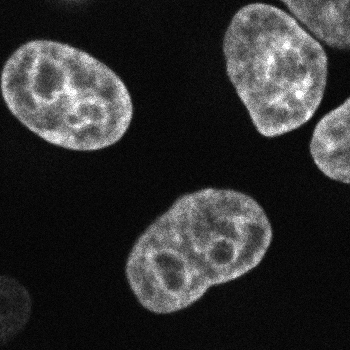

Supplement: Supplementary file 9 — Source data Fig. 3 [file 44318_2025_400_MOESM9_ESM.zip › Figure 3/3A/2023601_C2_2-HeLa-DMSO.tif]

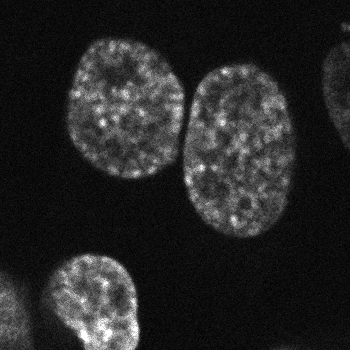

Supplement: Supplementary file 9 — Source data Fig. 3 [file 44318_2025_400_MOESM9_ESM.zip › Figure 3/3A/2023601_C3_1-HeLa-BI2536.tif]

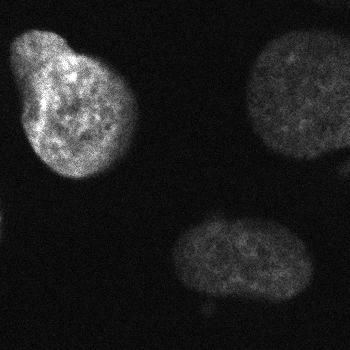

Supplement: Supplementary file 9 — Source data Fig. 3 [file 44318_2025_400_MOESM9_ESM.zip › Figure 3/3A/2023601_D2_1-U2OS-DMSO.tif]

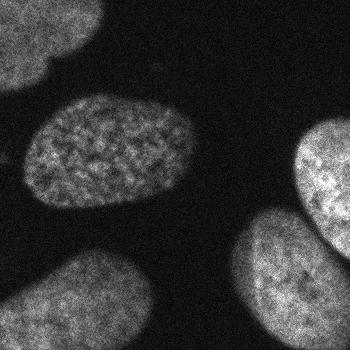

Supplement: Supplementary file 9 — Source data Fig. 3 [file 44318_2025_400_MOESM9_ESM.zip › Figure 3/3A/2023601_D4_1-U2OS-BI2536.tif]

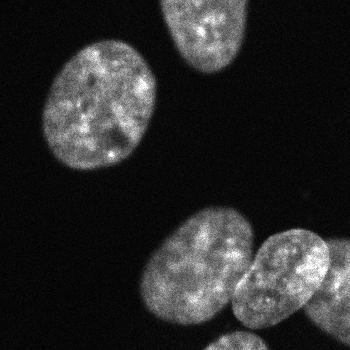

Supplement: Supplementary file 9 — Source data Fig. 3 [file 44318_2025_400_MOESM9_ESM.zip › Figure 3/3A/2023601_E2_1-RPE-DMSO.tif]

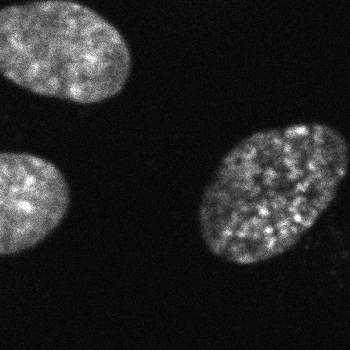

Supplement: Supplementary file 9 — Source data Fig. 3 [file 44318_2025_400_MOESM9_ESM.zip › Figure 3/3A/2023601_E4_1-RPE-BI2536.tif]

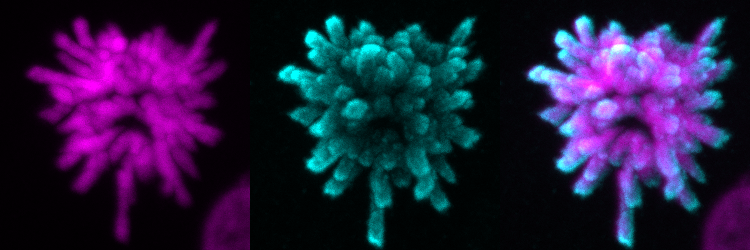

Supplement: Supplementary file 9 — Source data Fig. 3 [file 44318_2025_400_MOESM9_ESM.zip › Figure 3/3D/X_250_polophenotzpe_32slicesmax_H3S10ph_10umSB.tif]

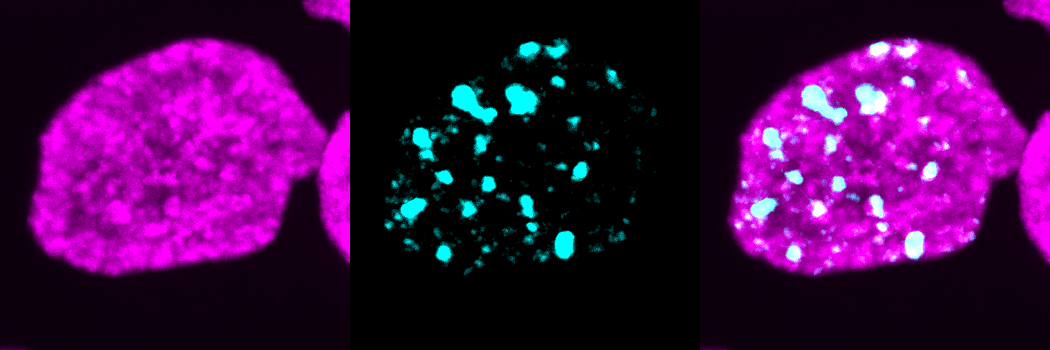

Supplement: Supplementary file 9 — Source data Fig. 3 [file 44318_2025_400_MOESM9_ESM.zip › Figure 3/3D/X_Ctrl_earlyprophase_H3S10ph_23slicesmidcellmax_10umSB.tif]

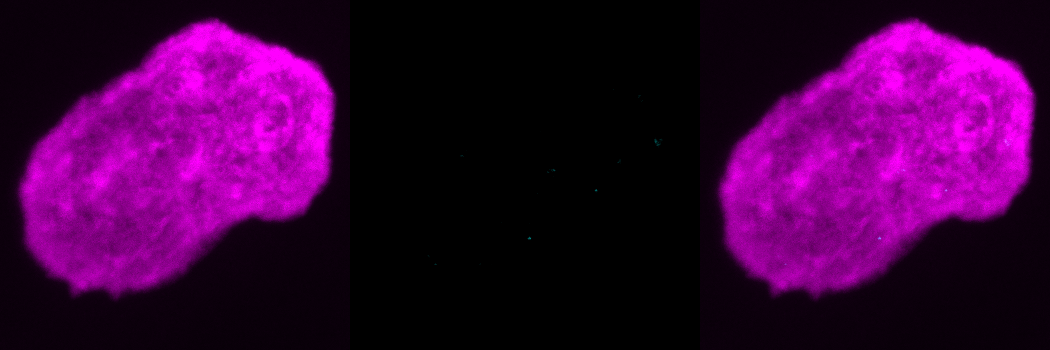

Supplement: Supplementary file 9 — Source data Fig. 3 [file 44318_2025_400_MOESM9_ESM.zip › Figure 3/3D/X_Ctrl_interphase_H3S10ph_32slicesmidcellmax_10umSB.tif]

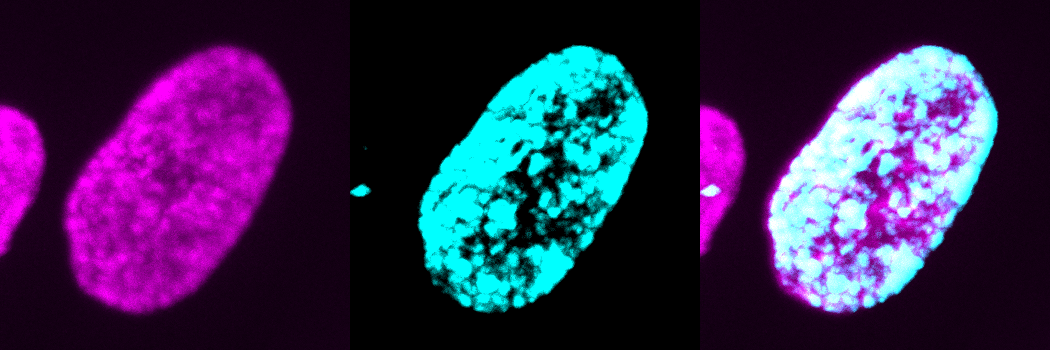

Supplement: Supplementary file 9 — Source data Fig. 3 [file 44318_2025_400_MOESM9_ESM.zip › Figure 3/3D/X_Ctrl_latepro_H3S10ph_24slicesmidcellmax_10umSB.tif]

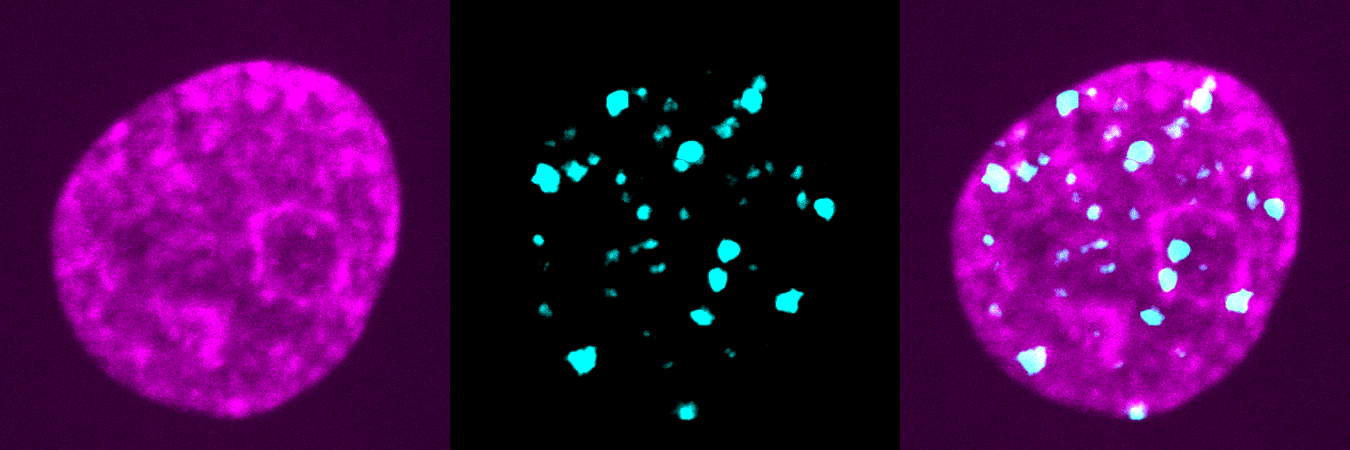

Supplement: Supplementary file 9 — Source data Fig. 3 [file 44318_2025_400_MOESM9_ESM.zip › Figure 3/3D/Y_250_propro_H3S10ph_13slicesmidcellmax_10umSB.tif]

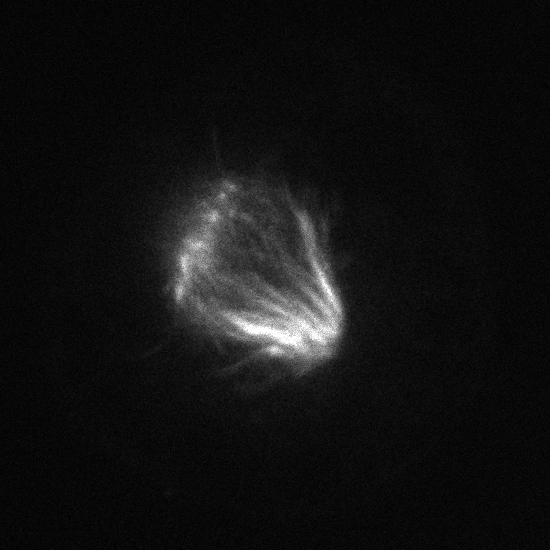

Supplement: Supplementary file 10 — Source data Fig. 4 [file 44318_2025_400_MOESM10_ESM.zip › Figure 4/4A/20220803_HeLaKyotoEB3eGFP_MTDynamics_Z3_1_DMSO-prometaphase.tif]

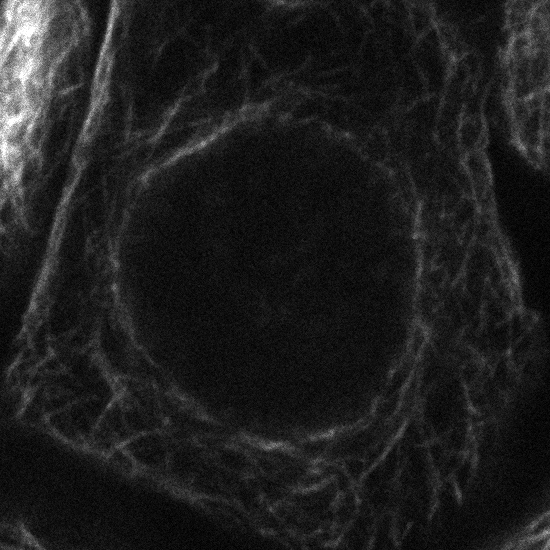

Supplement: Supplementary file 10 — Source data Fig. 4 [file 44318_2025_400_MOESM10_ESM.zip › Figure 4/4A/20220803_HeLaKyotoEB3eGFP_MTDynamics_Z3_2_DMSO-prophase.tif]

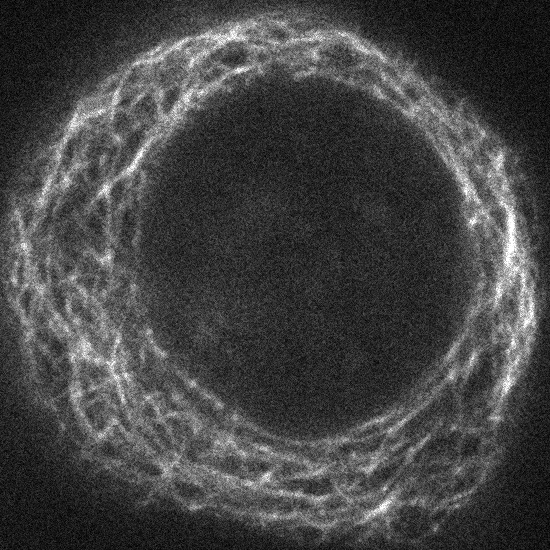

Supplement: Supplementary file 10 — Source data Fig. 4 [file 44318_2025_400_MOESM10_ESM.zip › Figure 4/4A/20220803_HeLaKyotoEB3eGFP_MTDynamics_Z4_3_BI2536-prolongedprophase.tif]

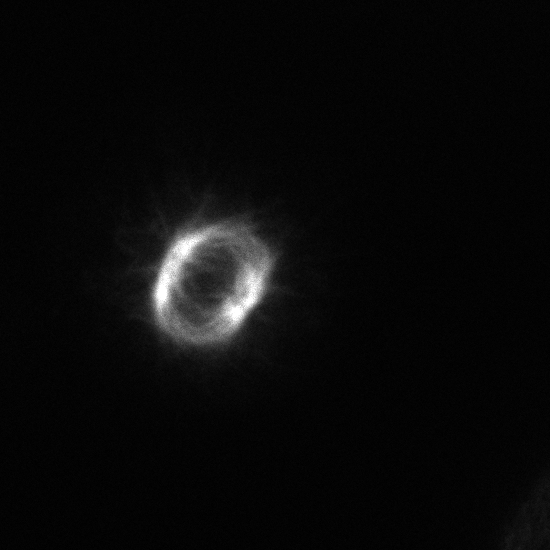

Supplement: Supplementary file 10 — Source data Fig. 4 [file 44318_2025_400_MOESM10_ESM.zip › Figure 4/4A/20220803_HeLaKyotoEB3eGFP_MTDynamics_Z4_7_BI2536-polo.tif]

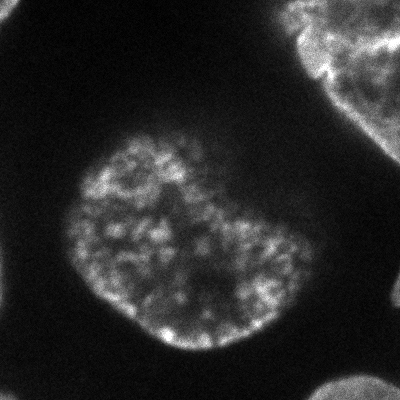

Supplement: Supplementary file 10 — Source data Fig. 4 [file 44318_2025_400_MOESM10_ESM.zip › Figure 4/4B/example image.tif]

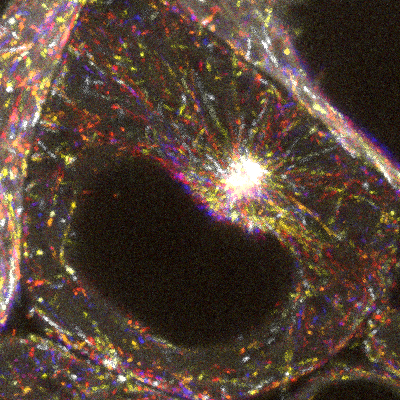

Supplement: Supplementary file 10 — Source data Fig. 4 [file 44318_2025_400_MOESM10_ESM.zip › Figure 4/4B/MAX_colored.tif]

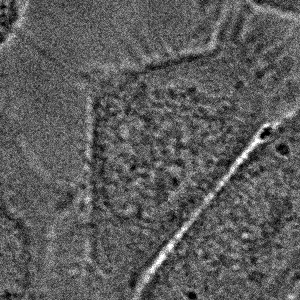

Supplement: Supplementary file 10 — Source data Fig. 4 [file 44318_2025_400_MOESM10_ESM.zip › Figure 4/4D/C3-20220705_D6_1-1-2.tif]

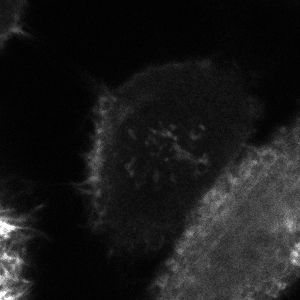

Supplement: Supplementary file 10 — Source data Fig. 4 [file 44318_2025_400_MOESM10_ESM.zip › Figure 4/4D/MAX_20220705_D6_1-1-2.tif]

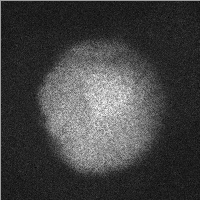

Supplement: Supplementary file 10 — Source data Fig. 4 [file 44318_2025_400_MOESM10_ESM.zip › Figure 4/4H/BI2536-MAX_20220927_D6_1-4-1.tif]

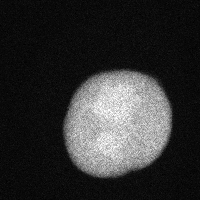

Supplement: Supplementary file 10 — Source data Fig. 4 [file 44318_2025_400_MOESM10_ESM.zip › Figure 4/4H/DMSO-MAX_20220927_D5_2-1-1.tif]
