# Supplementary material for: PLK1 inhibition delays mitotic entry revealing changes to the phosphoproteome of mammalian cells early in division
Source: EMBO J. 2025 Mar 3;44(7):1891–920. doi: 10.1038/s44318-025-00400-9 (PMC11962124; doi:10.1038/s44318-025-00400-9)
Supplement: Supplementary file 12 — Expanded View Figures [file 44318_2025_400_MOESM12_ESM.pdf]

Expanded View Figures

Figure EV1. Detailed characterization of the delayed mitotic entry in plk1-inhibited cells.

(**A**) HeLa cells expressing PCNA-mEGFP revealing S-phase progression after thymidine release. (**B**) Quantification of the S-phase duration in DMSO ($n = 20$) and BI 2536-treated ($n = 17$) cells. Unpaired Student's *t* test with Welch's correction was used for statistical significance assessment. n.s. corresponds to $p > 0.05$ ($p = 0.4028$). (**C**) Quantification of the duration from S-phase termination to NEBD and with and without thymidine synchronization in HeLa Kyoto cells measured by PCNA labeling. Unpaired Student's *t* test with Welch's correction between DMSO ($n = 15$) and BI2536-treated ($n = 15$) with added thymidine, and DMSO ($n = 30$) and BI 2536-treated ($n = 17$) without adding thymidine was used for statistical significance assessment. **** corresponds to $p < 0.0001$, with added thymidine $p = 7.98E{-}05$ and without added thymidine $p = 1.35E{-}08$. (**D**) Immunofluorescence images of HeLa Kyoto cells stained for γ-H2AX antibodies. Example images (left) and quantification of the number of γ-H2AX foci per nuclei (right) are shown. The DMSO ($n = 27$) and BI2536-treated ($n = 14$) cells were analyzed 8 and 17 h after thymidine release, respectively. Unpaired Student's *t* test with Welch's correction was used for statistical significance assessment. n.s. corresponds to $p > 0.05$ ($p = 0.5467$). (**E**) Model network modified from Rata et al, 2018 to include a plk1 dependent term. (**F**) Immunoblot analysis of samples collected at different time points after thymidine release and DMSO/BI2536 treatment. The cells were split after shaking off into supernatant (mitotic) and attached (interphasic) fractions. The blots were imaged after treatment with fluorescently-labeled secondary antibodies specific to the primary antibodies shown. Cdk1 was used as a loading control. For (**B**), (**C**), and (**D**): significance testing was done using unpaired Student's *t*-test with Welch's correction. For (**A**) and (**D**): scale bars are 10 µm. (**G**) Bifurcation graph of the mathematical model. Shown is the steady state active [cdk1-cyclin B] ($[CycBCdk1]$) as function of [cdk2-cyclin A] activity ($[CycACdk2_{Tot}]$). Stable and unstable steady states are indicated by a solid and dashed line, respectively. When cdk2-cyclin A activity is above the saddle node (SN) bifurcation only the high cdk1 activity steady state remains.

 © The Author(s)

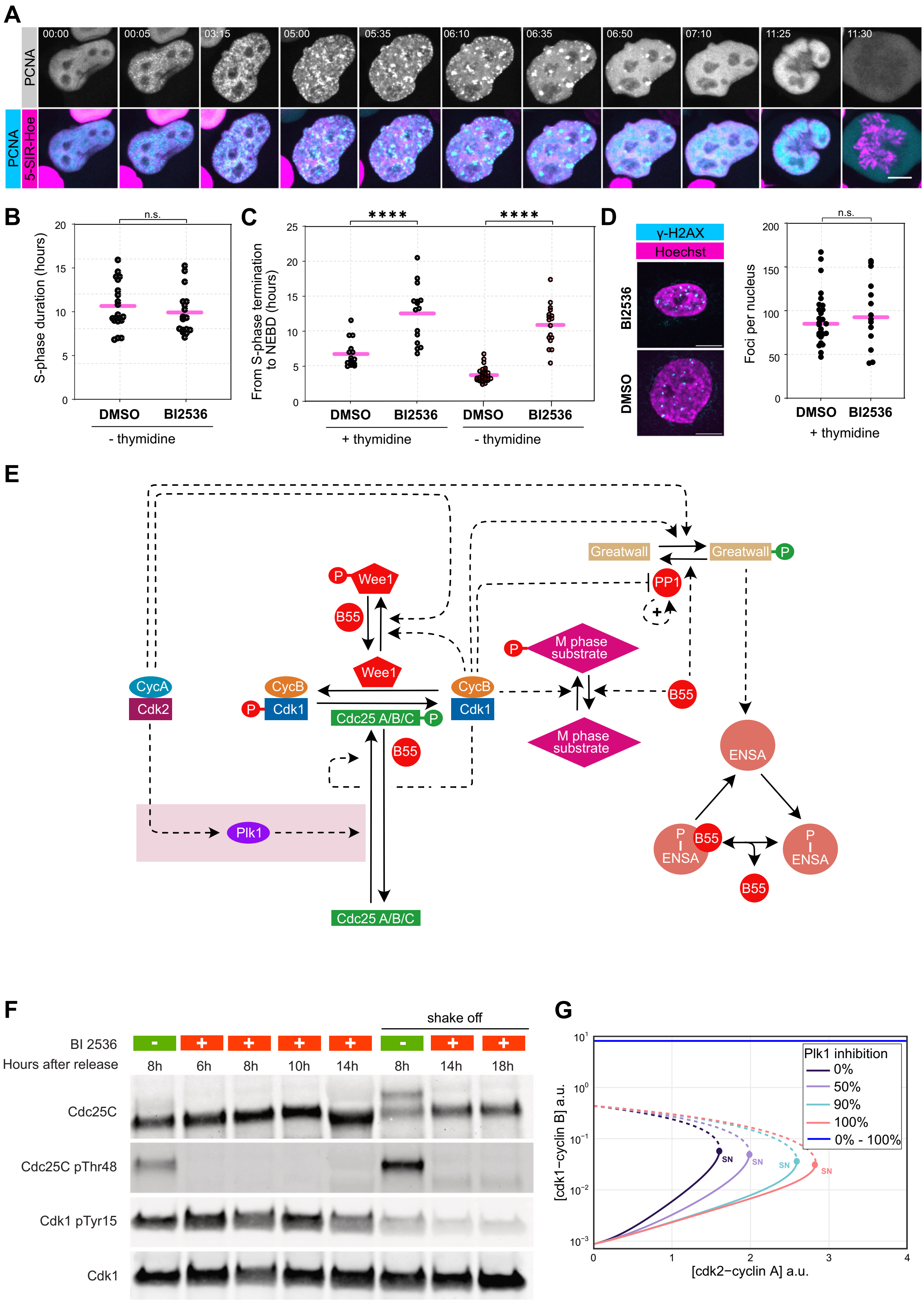
A
PCNA
PCNA 5-SIR-Hoe
00:00
00:05
03:15
05:00
05:35
06:10
06:35
06:50
07:10
11:25
11:30
B
S-phase duration (hours)
n.s.
DMSO
BI2536
- thymidine
C
From S-phase termination to NEBD (hours)
****
DMSO
BI2536
+ thymidine
- thymidine
D
γ-H2AX
Hoechst
BI2536
DMSO
Foci per nucleus
n.s.
+ thymidine
E
Greatwall
P
PP1
Wee1
B55
M phase substrate
CycA
Cdk2
CycB
Cdk1
Cdc25 A/B/C
Plk1
ENSA
F
BI 2536
Hours after release
shake off
8h
6h
10h
14h
18h
Cdc25C
Cdc25C pThr48
Cdk1 pTyr15
Cdk1
G
[cdk1−cyclin B] a.u.
[cdk2−cyclin A] a.u.
Plk1 inhibition
0%
50%
90%
100%
0% - 100%
SN

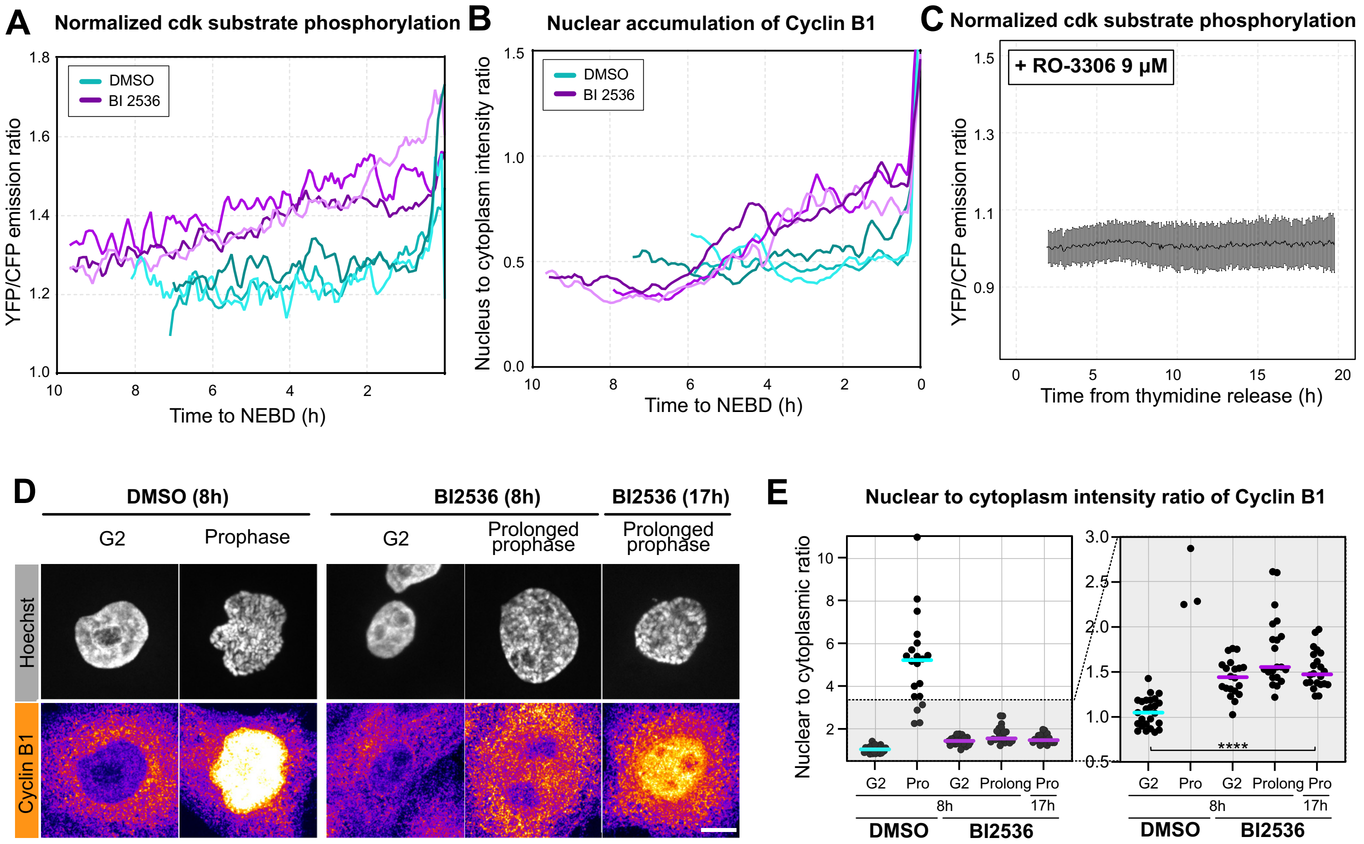

Figure EV2. Cdk1 activity and Cyclin B1 localization quantified in individual live cells and in fixed cells.

(**A**) Quantification of FRET ratio changes of the Eevee-spCDK FRET probe in individual control and BI 2536-treated cells. (**B**) Quantification of Cyclin B1 nucleus to cytoplasm intensity ratio in individual control and BI 2536-treated cells. (**C**) Quantification of FRET ratio changes of the Eevee-spCDK FRET probe in RO 3306-treated population of cells ($n = 59$). Mean ± Standard Deviation (SD) is shown. (**D**) Immunofluorescence of cyclin B1 in control and BI 2536-treated cells fixed at different time points after thymidine release. Scale bar is 10 µm. (**E**) Quantification of nuclear to cytoplasmic ratio of fluorescence intensity of cyclin B1 in control (G2 ($n = 25$) and prophase ($n = 20$)), and treated cells (G2 ($n = 20$), prolonged prophase ($n = 22$) 8 h after thymidine release and prolonged prophase ($n = 22$) 17 h after thymidine release) on images similar to (**D**). Inset magnifying the lower range is shown on the right panel. Unpaired Student's *t* test with Welch's correction was used for statistical significance assessment with **** corresponding to $p < 0.0001$ ($p = 2.22E{-}10$). Source data are available online for this figure.

 © The Author(s)

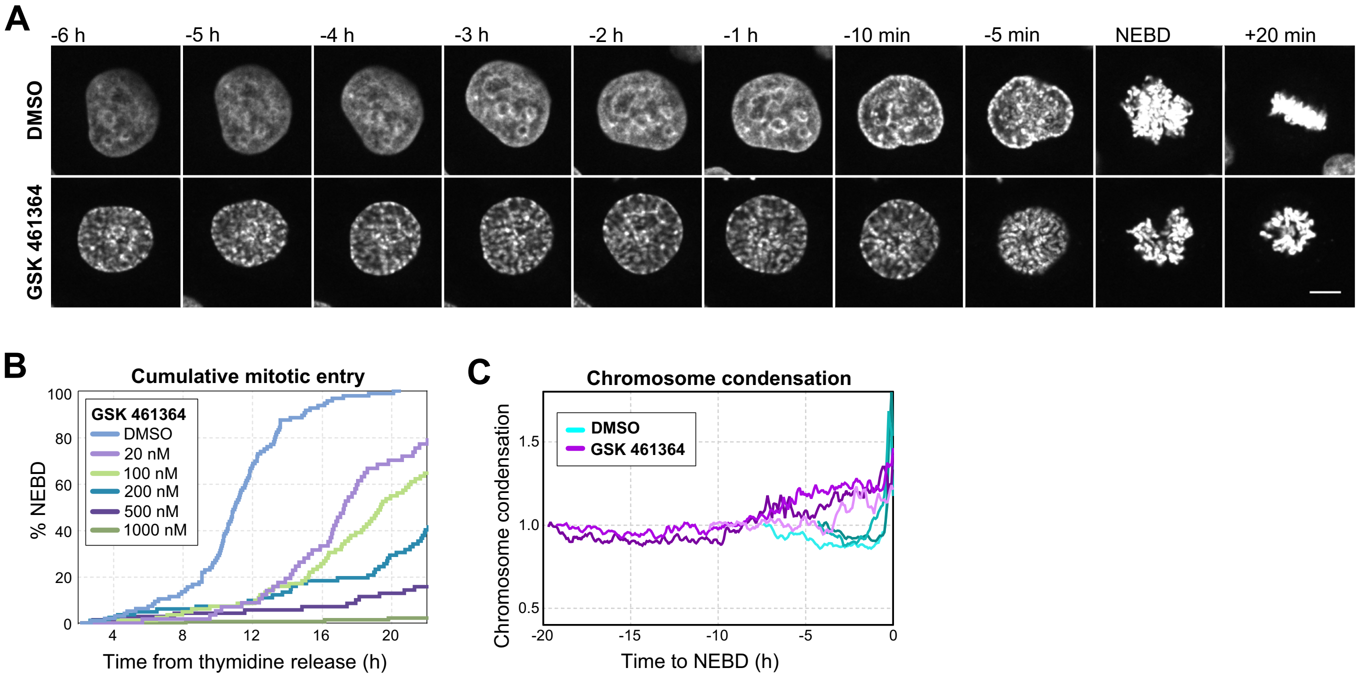

Figure EV3. A structurally unrelated Plk1 inhibitor recapitulates the phenotype caused by BI 2536.

(**A**) Montages of HeLa cells showing mitotic entry of exemplary control and GSK 461364-treated cells stained with 5-SiR-Hoechst. Time is relative to NEBD. Scale bar is 10 μm. (**B**) Cumulative plots of NEBD timing in response to increasing concentrations of GSK 461364 in HeLa Kyoto cells. (**C**) Quantification of chromosome condensation by standard deviation of fluorescence intensity of individual HeLa Kyoto cells on recording similar to shown in (**A**).

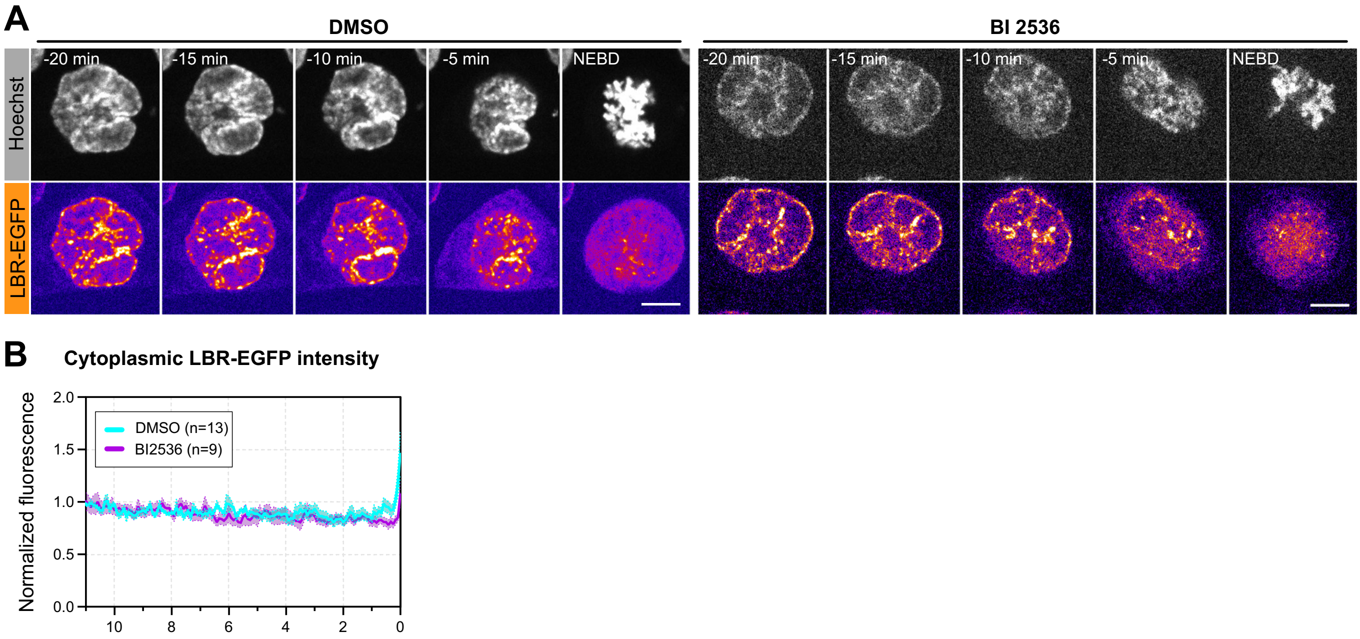

Figure EV4. Release kinetics of LBR, a protein of the nuclear envelope, is unaffected by Plk1 inhibition.

(**A**) Selected frames from a time lapse showing the localization of LBR-EGFP in the time leading to NEBD in DMSO and BI 2536-treated cells. Scale bar is 10 µm. (**B**) Quantification of LBR-EGFP mean cytoplasmic fluorescence intensity in DMSO and BI 2536-treated cells on recordings similar to (**A**). Data are normalized to the mean intensity in the first 5 frames of imaging. Mean ± standard error of mean (SEM) is shown.

© The Author(s)

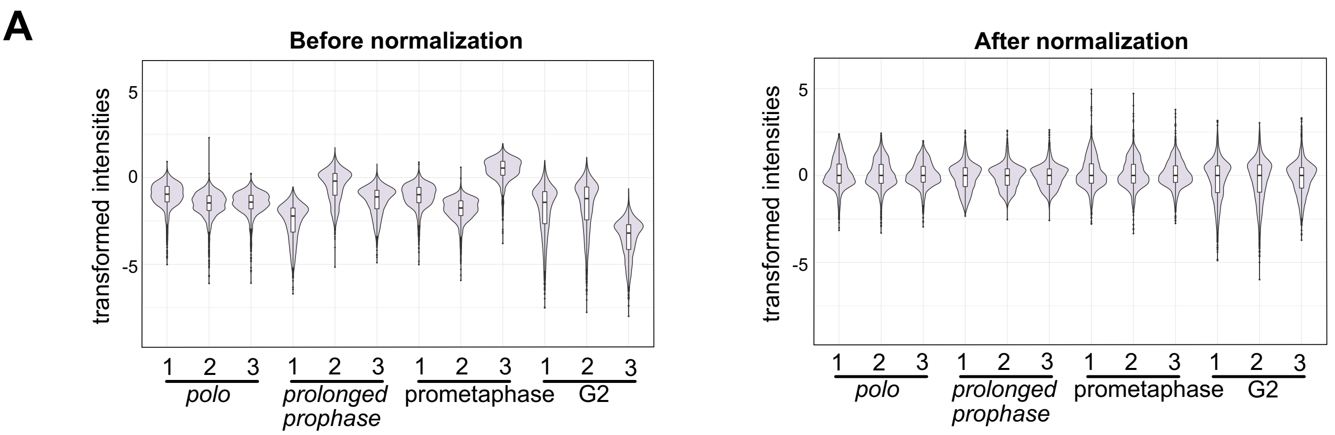

B Differentially phosphorylated proteins between *polo* and Prometaphase samples

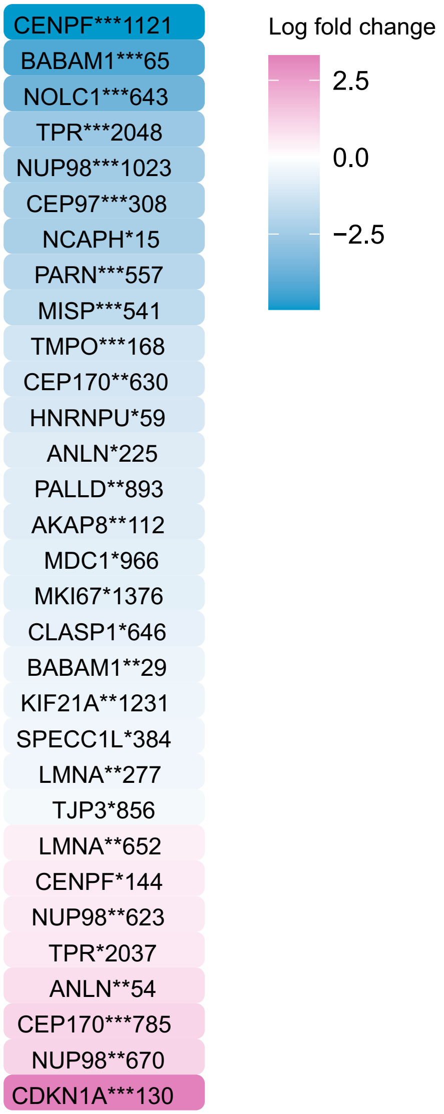

C Kinases negatively enriched in *polo* sample relative to prometaphase

Kinase	Kinase Group	Log2 Enrichment	Significance (-log adjusted p value)
PLK1	Other	-2.9989	8.884269
CAMK2D	CAMK	-2.05404	5.461809
PLK4	Other	-2.23991	2.778463
MAPKAPK3	CAMK	-2.06998	2.547787
MAPKAPK2	CAMK	-1.55397	2.547787
CHK1	CAMK	-1.81409	2.524474
GCN2	Other	-1.91386	2.524474
DLK	TKL	-2.1509	2.334321
PKN3	AGC	-2.44636	2.302078
CAMK4	CAMK	-2.61347	2.247387

D Kinases enriched in prolonged prophase

Kinase	Kinase Group	Log2 Enrichment	Significance (-log adjusted p value)
KIS	Other	0.924796	4.592543
CDK5	CMGC	0.719101	2.568039
CDK2	CMGC	0.645396	2.357501
JNK3	CMGC	0.732357	2.357501
CDK8	CMGC	0.660207	2.234585
CDK13	CMGC	0.643014	1.462511
NLK	CMGC	0.698971	1.362713
CDK12	CMGC	0.637616	1.362713
CDK16	CMGC	0.557815	1.362713
CDK1	CMGC	0.5435	1.362713

© The Author(s)

Figure EV5. Kinase profiling confirms specific inhibition of plk1 by BI 2536, and cdks as the primary kinases active in the prolonged prophase state.

(**A**) Violin plots showing the distribution of the log(2) transformed intensities of the phosphosites detected in the 12 samples (4 samples in 3 replicates) before and after normalization. For the shown box plots, the center corresponds to the median and the lower and upper hinges correspond to the first and third quartiles (the 25th and 75th percentiles). The upper and lower whisker extend from the hinge to the largest and smallest value, respectively, no further than 1.5 * IQR from the hinge (where IQR is the inter-quartile range, or distance between the first and third quartiles). Data beyond the end of the whiskers are outliers and are plotted individually. (**B**) Differentially phosphorylated peptides in *polo* cells in comparison to prometaphase cells that were detected using linear modeling. The gene names of the corresponding peptides are shown, the asterisks mark the adjusted *p*-value with ***, **, * corresponding to $p \leq 0.001$, 0.01, and 0.05, respectively, followed by the position of the phosphosite within the amino acid sequence of the protein. The color coding corresponds to the log(2) fold change of intensity values. Statistical significance was assessed using a moderated *t*-test where empirical Bayes moderation was applied to stabilize variance estimates across genes. (**C**) List of kinases least enriched in the polo sample in comparison to the prometaphase sample after applying linear modeling. (**D**) List of kinases most enriched in the prolonged prophase sample after applying linear modeling. For (**C**) and (**D**), the enrichments were determined using one-sided exact Fisher's tests and corrected for multiple hypotheses using the Benjamini–Hochberg method.

 © The Author(s)
